# Numerical Analysis of Hydrodynamic Flow in Microfluidic Biochip for Single-Cell Trapping Application

**DOI:** 10.3390/ijms161125987

**Published:** 2015-11-09

**Authors:** Amelia Ahmad Khalili, Mohd Ridzuan Ahmad

**Affiliations:** 1 Department of Control and Mechatronic Engineering, Faculty of Electrical Engineering, Universiti Teknologi Malaysia, Skudai, Johor 81310, Malaysia; amelia.ahmadkhalili@gmail.com; 2 Institute of Ibnu Sina, Universiti Teknologi Malaysia, Skudai, Johor 81310, Malaysia

**Keywords:** microfluidic, hydrodynamic, single-cell trapping, finite element

## Abstract

Single-cell analysis has become the interest of a wide range of biological and biomedical engineering research. It could provide precise information on individual cells, leading to important knowledge regarding human diseases. To perform single-cell analysis, it is crucial to isolate the individual cells before further manipulation is carried out. Recently, microfluidic biochips have been widely used for cell trapping and single cell analysis, such as mechanical and electrical detection. This work focuses on developing a finite element simulation model of single-cell trapping system for any types of cells or particles based on the hydrodynamic flow resistance (Rh) manipulations in the main channel and trap channel to achieve successful trapping. Analysis is carried out using finite element ABAQUS-FEA™ software. A guideline to design and optimize single-cell trapping model is proposed and the example of a thorough optimization analysis is carried out using a yeast cell model. The results show the finite element model is able to trap a single cell inside the fluidic environment. Fluid’s velocity profile and streamline plots for successful and unsuccessful single yeast cell trapping are presented according to the hydrodynamic concept. The single-cell trapping model can be a significant important guideline in designing a new chip for biomedical applications.

## 1. Introduction

Lab on a Chip (LOC) and Micro Total Analysis Systems (μTAS) have attracted researchers’ attention in the areas of biotechnology and biomedical engineering. The rise in interest is due to the utilization of these devices in a broad range of biological and biomedical application areas including genomics, enzymatic analysis, disease diagnosis, cell treatment, drug screening, single-cell analysis, and drug delivery. In cellular biology, single-cell analysis refers to the study of individual cells isolated from tissues in multi-cellular organisms. Conventionally, cell analyses are conducted with large populations of cells and data measurement can only represent the average values summed over the responses of many cells. Therefore, single-cell analysis is important to obtain more precise information and to reveal the properties of individual cells and cell-to-cell differences [1].

In order to perform single-cell analysis in microfluidic devices, trapping of a single cell is necessary. A variety of techniques have been employed to trap an individual cell. For example, microwell-based [2,3,4,5,6], dielectrophoresis-based [7,8,9,10,11], and hydrodynamic-based [12,13,14,15,16,17,18,19,20,21,22,23,24] microfluidic devices for single-cell trapping have been developed in response to an increasing demand for simple yet reliable tools for high-throughput cell manipulation at the single cell level. In microwell-based platforms, a precise geometry design is required to achieve a high trapping efficiency [4]. Dielectrophoresis-based cell trapping applied a non-uniform AC field to manipulate polarized particles in suspension and is an effective technique to efficiently manipulate a single cell. However, it appears to damage the trapped cells, thus affecting the cell proliferation. Hydrodynamic trapping uses the altered fluidic resistance created by microstructures on a fluid path, such as sieve-like traps [23,24,25] or small trapping sites [12,13,14,15,16,17,26,27], to control the movement of cells in a microchannel. For straight or serpentine-shaped channels with trapping sites, the fluidic resistances of these channels are carefully calculated so that the fluid and cells in the main channel will preferentially flow into the trapping sites when they are empty, but bypass them when they are occupied with a cell. The main challenge in hydrodynamic trapping is that it requires a precise microfluidic control of multiple streams. Further investigation and optimization of cells’ trapping efficiencies are still required [20].

The concept of hydrodynamic trapping for small trapping sites was originally proposed by Tan and Takeuchi [26]. However, a proof of concept is performed by experimental work only and no prior optimization of the microfluidic design through simulation works has been reported. From our point of view, this could probably involve high fabrication costs and it might be time consuming to find the optimized geometry through devices fabricated by trial and error. Therefore, our work is focused on developing the single-cell trapping model to produce a finite element simulation system that could be used to optimize a channel’s geometry for any type of cells or particles. The model is developed based on hydrodynamic flow resistance (*Rh*) manipulation in the main channel and trap channel to achieve successful trapping. This study provides a proof of concept demonstration for a cell positioning platform to trap single cells and a guideline for designing and optimizing single-cell trapping channel is proposed. The example of a thorough optimization study is presented using a 5-μm yeast cell model. Microchannels’ geometrical size optimization is carried out by manipulating the geometry of the trap channel, trap hole, and main channel. Numerical simulations are conducted to evaluate the cells’ trapping efficiencies for a variety of geometrical parameters. Fluid’s velocity profile and streamline plots are studied to explain the fluid’s stream direction according to the hydrodynamic principles. The single-cell trapping system is dependent on the cell’s size, as different cells require different optimized trapping channel sizes, trap hole’s sizes, and main channel lengths (*L*_Main_). Therefore, it is important for us to optimize the channel’s geometry before fabricating the real device to reduce time and fabrication costs.

## 2. Results and Discussion

### 2.1. Verification of Hydrodynamic Trapping Concept

**Figure 1:**
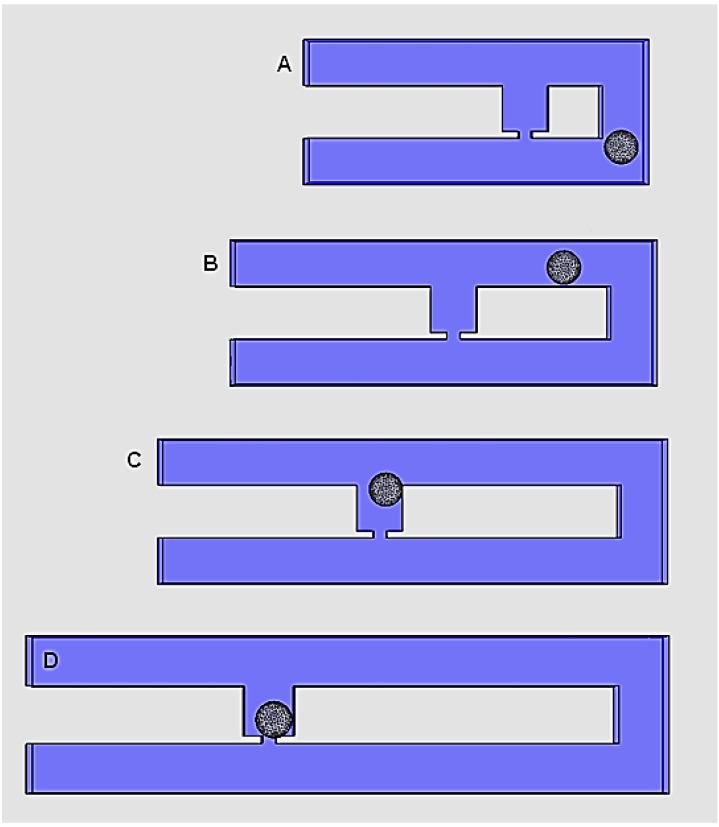
Cell trapping results at simulation time of 28 s for cell trapping model with *W*_Hole_ of 2 μm for different *Rh*_Main_/*Rh*_Trap_ ratio of (**A**) 1.5; (**B**) 2.5; (**C**) 3.5; and (**D**) 4.5.

The purpose of this finite element analysis is to verify the hydrodynamic trapping concept in the proposed model and to perform geometry optimization for efficient single-cell trapping. According to the hydrodynamic trapping concept proposed by Tan and Takeuchi [26], single-cell/particle trapping is achievable when the flow rate of trap channel to main channel (*Q*_Trap_/*Q*_Main_) ratio is above 1. To verify the concept, the cell trapping model with trap hole’s width (*W*_Hole_) 2.0 μm is used to study the appropriate flow resistance of main channel to trap channel (*Rh*_Main_/*Rh*_Trap_) ratio. Main channel’s length (*L*_Main_) is manipulated to create an *Rh*_Main_/*Rh*_Trap_ ratio ranging from 1 to 6. Increasing the *Rh*_Main_/*Rh*_Trap_ ratio is proportional with the increase in the main channel’s (loop path) length. A yeast cell model is successfully trapped when a *Rh*_Main_/*Rh*_Trap_ ratio of 3.5 or higher is used (Figure 1C,D). Furthermore, results show that an *Rh*_Main_/*Rh*_Trap_ ratio ranging between 1.0 and 3.0 caused the cell to bypass the trap channel (Figure 1A,B).

**Figure 2:**
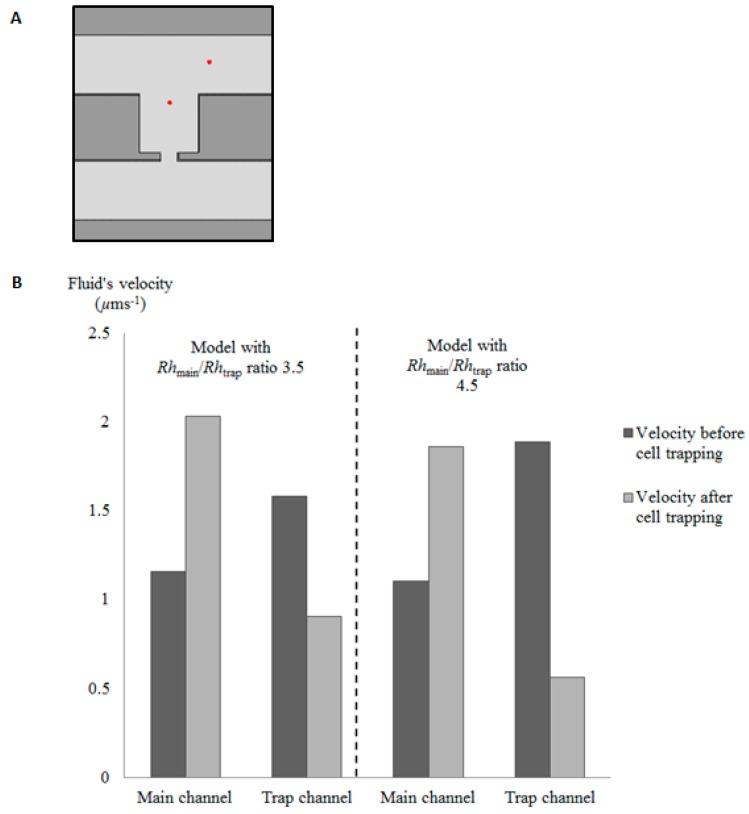
(**A**) Points representing velocity of fluid in the trap channel (**left** side) and main channel (**right** side); (**B**) graph representing velocity of fluid in trap channel and main channel for cell trapping model with *Rh*_Main_/*Rh*_Trap_ ratio of 3.5 or 4.5 before and after cell trapping.

From the simulation result, an *Rh*_Main_/*Rh*_Trap_ ratio of 3.5 or above is found to be able to trap single cells via the hydrodynamic trapping concept. To further verify the principle of the hydrodynamic trapping, the fluid’s velocity inside the main channel and trap channel before and after trapping is analyzed. The velocity of the fluid at two points is analyzed (Figure 2A) to represent the fluid’s velocity before and after trapping for a cell trapping model with an *Rh*_Main_/*Rh*_Trap_ ratio of 3.5 or 4.5 (Figure 2B). The fluid’s velocity in the main channel before cell trapping is found to be lower than the velocity in the trap channel (Figure 2B). However, after the cell is trapped inside the trap channel, the fluid’s velocity inside the trap channel decreases instantly and the fluid’s velocity at the main channel increases dramatically. This finding supports the principle of hydrodynamic trapping in which when the trapping side is empty, the trap channel will have lower flow resistance compared to the bypass channel (main channel). When the velocity of fluid in the trap channel is higher, it leads to a lower hydrodynamic resistance in the trapping site, which creates a trapping stream that will direct cells into the trap channel. When a cell has been trapped inside the trap channel, it blocks the trap hole and drastically decreases the fluid’s velocity inside the trap channel. The direction of fluid flow diverges from the trap channel to the loop path (main channel). Therefore, subsequent cells will be directed to the loop path. The simulation results are found to be in good agreement with the reported experimental results.

### 2.2. Effects of Rh_Main_/Rh_Trap_ Ratio in Cell Trapping

Subsequent simulation is carried out to study the effects of the *Rh*_Main_/*Rh*_Trap_ ratio on cell trapping using a model with trap hole widths of 1.0 or 1.5 μm. The main channel’s length has to be manipulated to comply with the desired *Rh*_Main_/*Rh*_Trap_ ratio. Similar trapping behavior is obtained when the *W*_Hole_ is decreased to 1.5 μm. The hydrodynamic concept works accordingly and the yeast cell is able to be directed towards the trap channel by the fluid stream when the *Rh*_Main_/*Rh*_Trap_ ratio is 3.5 and above. However, for models with a trap holes width of 1.0 μm, a cell is not able to be trapped even though the *Rh*_Main_/*Rh*_Trap_ ratio is above 3.5. The cell is found to not be moving to the trap channel and bypasses it (data not shown). This result shows that a *W*_Hole_ of 1 μm is not suitable for the specified trap channel dimension (7 μm width, height, and length). The design fails to follow the hydrodynamic trapping concept, probably due to the small trap hole (<1/5 of trap channel’s width (*W*_Trap_)). The small *W*_Hole_ probably cause a very low fluid velocity distribution and produce low pressure drop that unable to capture cells into the trap channel. [28]. A simulation study performed by Khalili *et al.* [28] showed the same trend of results when a very small *W*_Hole_/*W*_Trap_ is used (<1/5). For designing a single-cell trapping channel, we suggest for the *W*_Hole_ to be more than 1/5 of *W*_Trap_ for a uniform *H*_Channel_. Table 1 summarizes the single-cell trapping model’s ability for different *W*_Hole_, *H*_Channel_, and trap channel’s length (*L*_Trap_), and various *Rh*_Main_/*Rh*_Trap_ ratios.

**Table 1:** Cell trapping results for different single-cell trapping model with different sizes of *W*_hole_, *H*_chan_, and *L*_Trap_ and various *Rh*_Main_/*Rh*_Trap_.

Ratio of *Rh*_Main_/*Rh*_Trap_	Ability to Trap Cells													
	*W*_Hole_: 1.0 μm	*W*_Hole_: 1.5 μm	*W*_Hole_: 2.0 μm	*W*_Hole_: 2.5 μm	*W*_Hole_: 3.0 μm	*W*_Hole_: 3.5 μm	*H*_Chan_: 6.0 μm	*H*_Chan_: 7.0 μm	*H*_Chan_: 8.0 μm	*H*_Chan_: 9.0 μm	*L*_Trap_: 3.0 μm	*L*_Trap_: 5.0 μm	*L*_Trap_: 7.0 μm	*L*_Trap_: 9.0 μm
1.0	no	no	no	no	no	no	no	no	no	no	no	no	no	no
1.5	no	no	no	no	no	no	no	no	no	no	no	no	no	no
2.0	no	no	no	no	no	no	no	no	no	no	no	no	no	no
2.5	no	no	no	no	no	no	no	no	no	no	no	no	no	no
3.0	no	no	no	no	no	no	no	no	no	no	no	no	no	no
3.5	no	yes	yes	yes	yes	yes	yes	yes	yes	yes	yes	yes	yes	yes
4.5	no	yes	yes	yes	yes	yes	yes	yes	yes	yes	yes	yes	yes	yes
5.0	no	yes	yes	yes	yes	yes	yes	yes	yes	yes	yes	yes	yes	yes
5.5	no	yes	yes	yes	yes	yes	yes	yes	yes	yes	yes	yes	yes	yes
6.0	no	yes	yes	yes	yes	yes	yes	yes	yes	yes	yes	yes	yes	yes

Fixed geometry for *W*_Hole_ optimization: *H*_Chan_: 7 μm, *W*_Trap_: 7 μm, *L*_Trap_: 7 μm, *L*_Hole_: 1 μm; fixed geometry for *H*_Channel_ optimization: *W*_Trap_: 7 μm, *L*_Trap_: 7 μm, *W*_Hole_: 2 μm, *L*_Hole_: 1 μm; fixed geometry for *L*_Trap_ optimization: *H*_Chan_: 7 μm, *W*_Trap_: 7 μm, *L*_Trap_: 7 μm, *W*_Hole_: 2 μm.

**Figure 3:**
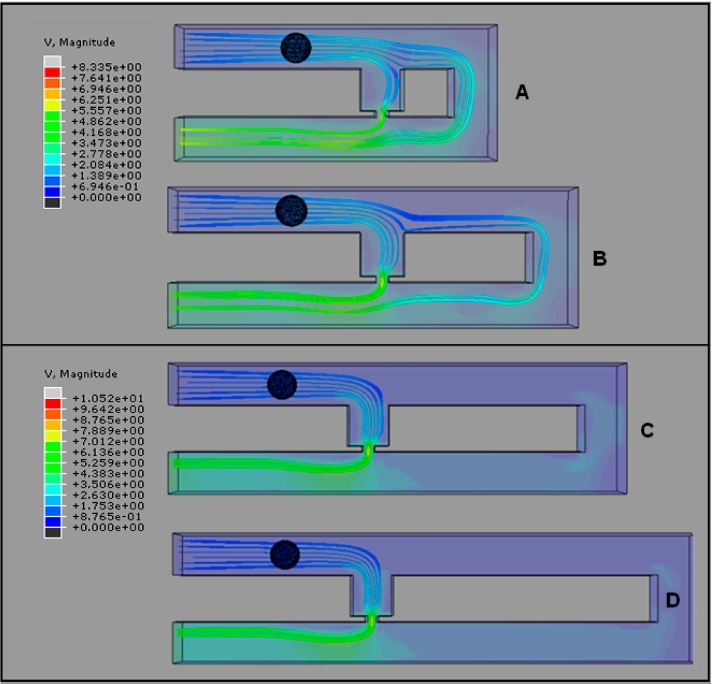
Velocity streamlines before cell trapping (top view) for cell trapping model with *W*_Hole_ of 2 μm for different *Rh*_Main_/*Rh*_Trap_ ratios of (**A**) 1.5; (**B**) 2.5; (**C**) 3.5; and (**D**) 4.5. V represents the fluid’s velocity in μms^−1^.

**Figure 4:**
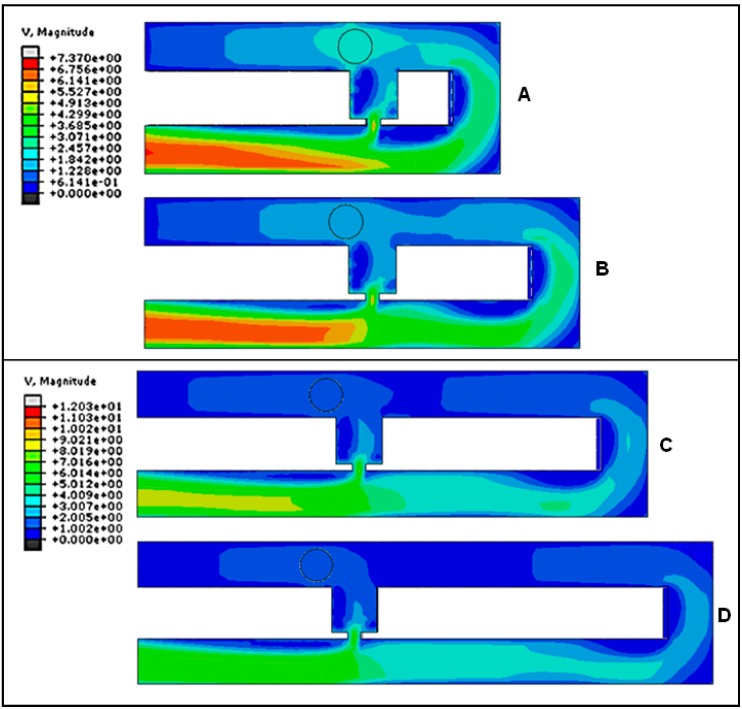
Velocity of fluid before cell trapping for single-cell trapping model with trapping hole width of 2 μm for *Rh*_Main_/*Rh*_Trap_ ratios of (**A**) 1.5; (**B**) 2.5; (**C**) 3.5; and (**D**) 4.5. V represents the fluid’s velocity in μms^−1^.

The fluid velocity profile and velocity streamline field of the cell trapping model are analyzed to understand the hydrodynamic trapping mechanism. Fluid velocity streamlines present the direction the fluid streams are heading, while velocity profiles represent the velocity value in the channel by the contour color. The velocity streamlines produced by the cell trapping model with an *Rh*_Main_/*Rh*_Trap_ ratio below 3.5 (Figure 3A,B), are found to be not fully directed to the trap channel and the portions of the streamlines that passed through the trap channel are directed to the loop. The produced fluid streams unable to direct the cell into the trap channel. This finding is in agreement with the fluid’s velocity distribution produced by the same model (Figure 4A,B). Results show that the main channel’s (loop path) fluid velocity for the single-cell trapping model with an *Rh*_Main_/*Rh*_Trap_ ratio of 1.5 and 2.5 is higher compared to the trap channel’s fluid velocity. Therefore the main stream will direct the yeast cell to flow into the main channel’s path and bypass the trap channel.

In contrast with the cell trapping model with an *Rh*_Main_/*Rh*_Trap_ ratio of 3.5 and above (Figure 3C,D), the streamlines profiles show the fluid flow diverging from the main channel to the trap channel and directed towards the trap channel. For models with an *Rh*_Main_/*Rh*_Trap_ ratio of 3.5 or 4.5 (Figure 4C,D), the fluid’s velocity distribution from the trap hole to the trap channel is higher compared to the fluid’s velocity in the main channel. These results show that the trap channel produces lower hydrodynamic resistance than the main channel and the mainstream will direct the yeast cell into the trap channel. Both models with an *Rh*_Main_/*Rh*_Trap_ ratio of 3.5 and 4.5 produce almost similar fluid velocity patterns that will produce appropriate pressure drop for the cell to be trapped.

**Figure 5:**
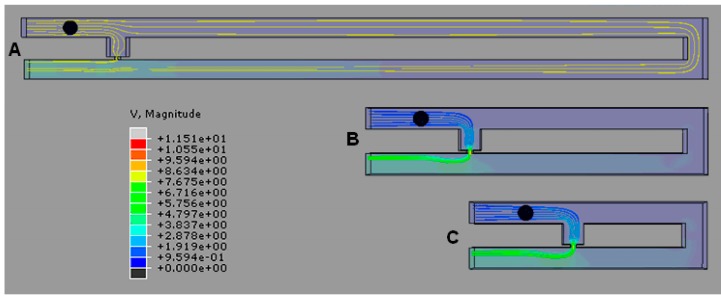
Velocity streamlines for the cell trapping model (top view) with *Rh*_Main_/*Rh*_Trap_ ratio of 3.5 for model with trap hole width of (**A**) 1.0 μm; (**B**) 1.5 μm; or (**C**) 2.0 μm. V represents the fluid’s velocity in μms^−1^.

The hydrodynamic trapping concept is found to be ineffective for a cell trapping model with a *W*_Hole_ of 1.0 μm. Subsequently, the *L*_Main_ has been increased to obtain an *Rh*_Main_/*Rh*_Trap_ ratio between 3.5 and 6.0; however, the cell trapping is not successful. The fluid velocity streamlines produced by this model show different profiles compared to the streamlines produced by models with *W*_Hole_ of 1.5 μm and 2.0 μm (Figure 5). The streamlines profile for the model shows that the flow direction is not fully focused into the trap channel but diverted to both the trap channel and the loop path directions (Figure 5A). The behavior of the fluid before cell bypass trap channel represents same trend of velocity profile and streamlines as models with unsuccessful trapping (Figure 3A,B). From the simulation results, the minimum main channel length needed to perform successful trapping is the length which produces an *Rh*_Main_/*Rh*_Trap_ ratio of 3.5 (with the exception of the model with *W*_Hole_ of 1.0 μm).

**Figure 6:**
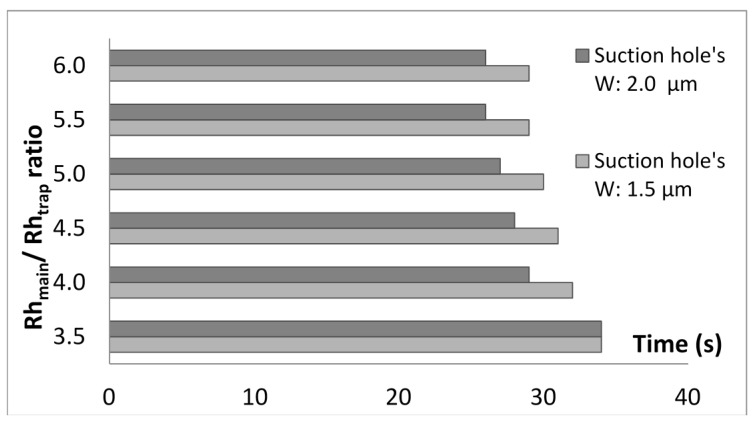
Cell trapping time for model with different *Rh*_Main_/*Rh*_Trap_ ranging from 3.5 to 6.0 for single-cell trapping model with trapping hole widths of 1.5 and 2.0 μm.

Both cell trapping models with a trap hole width of 1.5 μm or 2.0 μm are found to be able to trap the yeast cell model with almost similar velocity profile. However, there are variations in the complete cell trapping time (time when the cell touches the surface of the trap channel) between different *Rh*_Main_/*Rh*_Trap_ ratios. A higher ratio requires a shorter time for the trapping process compared to a lower ratio. The graph in Figure 6 shows the results of trapping time for cell trapping models with *W*_hole_ of 1.5 and 2.0 μm for *Rh*_Main_/*Rh*_Trap_ ranging from 3.5 to 6.0. From the graph, it is evident that the trapping time decreases with increasing *Rh*_Main_/*Rh*_Trap_. This is probably due to the higher *Rh*_Main_/*Rh*_Trap_ ratio being able to perform velocity distribution in a shorter time compared to the lower *Rh*_Main_/*Rh*_Trap_. A greater *Rh*_Main_/*Rh*_Trap_ ratio could provide a lower hydrodynamic resistance in the trap channel and could transfer the fluid at a faster rate. The velocity distribution produces different pressure from the main channel to the trap hole, making the flow resistance inside the trap channel lower than the main channel. Therefore, together with the fluid, cells will flow to the lower flow resistance area and be trapped. A bigger *W*_hole_ value is able to produce shorter trapping time compared to the smaller height. Analyses are conducted for *W*_hole_ of 2.5, 3.0, and 3.5 μm and similar results are obtained where the cell is able to be trapped with *Rh*_Main_/*Rh*_Trap_ ratio of 3.5 and above (refer Table 1).

### 2.3. Optimization of Trap Channel’s Length

**Figure 7:**
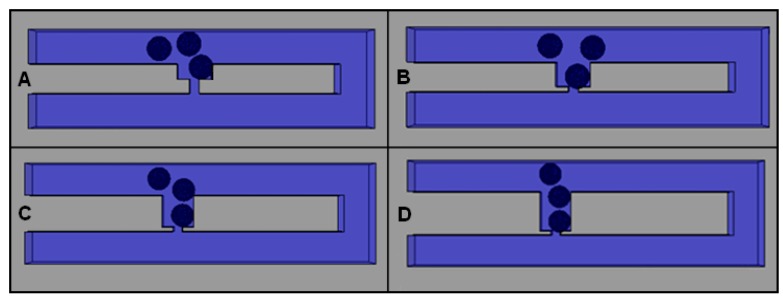
Cell trapping results for the optimization of different *L*_Trap_ values: (**A**) 3 μm; (**B**) 5 μm; (**C**) 7 μm; and (**D**) 9 μm.

After investigating the effects of *Rh*_Main_/*Rh*_Trap_ ratio for the single cell trapping model, the efficiency of the single-cell trapping is enhanced by optimizing the trap channel’s length (*L*_Trap_) (refer Figure 2A). Using yeast cell and four different *L*_Trap_, the behavior of cell trapping is observed. A model with *L*_Trap_ of 3 μm is able to trap single cells; however, after a cell is trapped, both of the paths to the loop and the outlet will eventually be blocked, causing clogging of subsequent cells at the main channel and thus preventing the smooth movement of cells towards the outlet (Figure 7A). Therefore the length is not suitable for efficient cell trapping. From the results, a model with *L*_Trap_ of 5 μm is found to be the most suitable length to trap a 5-μm yeast cell as it could allow the subsequent cell to flow to the loop and heading to the outlets (Figure 7B). For a model with *L*_Trap_ of 7 or 9 μm, results show that two cells are able to enter the trap channel during cell trapping (Figure 7C,D). The aim for the cell trapping model development is to trap a single cell; therefore the *L*_Trap_ of 7 and 9 μm are not suitable for efficient single-cell trapping.

**Figure 8:**
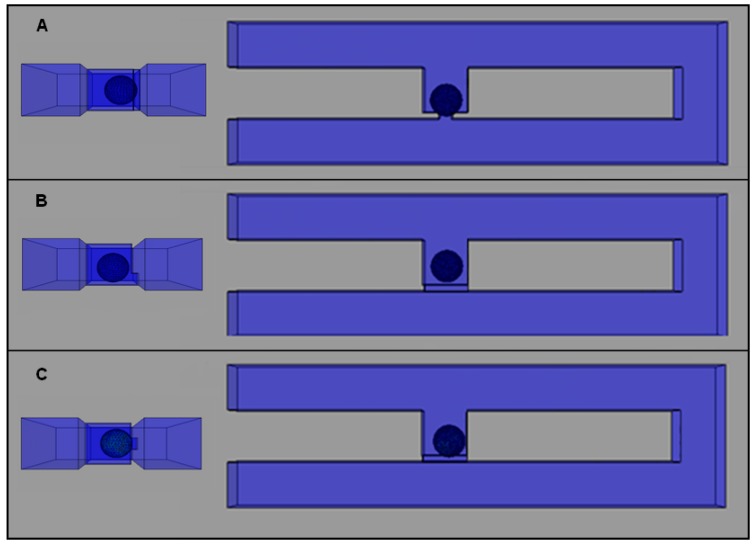
Cell trapping results at simulation time of 34s for cell trapping model with the same trap hole size (2, 7 and 7 μm of *W*, *L* and *H*, respectively) for an *Rh*_Main_/*Rh*_Trap_ ratio of 3.5 at three different positions: (**A**) model A; (**B**) model B; and (**C**) model C. (**Left**) side view; (**right**) top view of the model.

### 2.4. Effects of Different Trap Hole Positions

**Figure 9:**
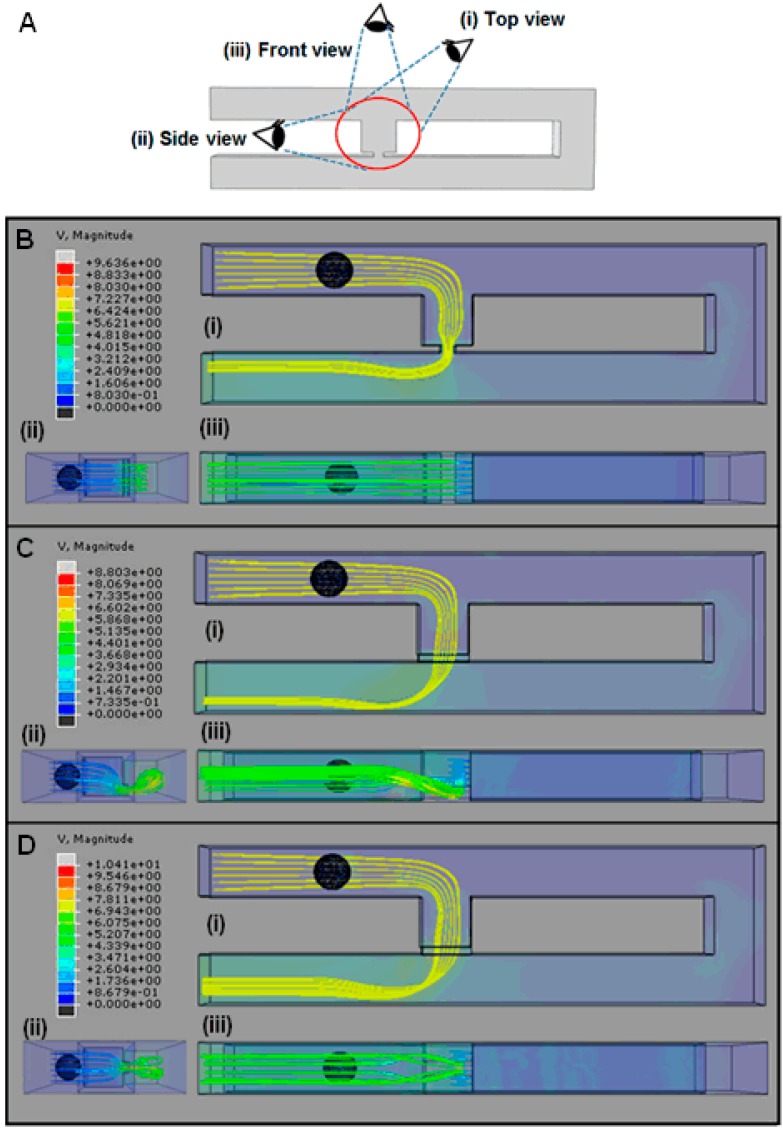
Streamline velocity for three different cell trapping models with the same trap hole size (2 μm, 7 μm, and 7 μm of *W*, *L*, and *H*, respectively) for an *Rh*_Main_/*Rh*_Trap_ ratio of 3.5 at three different positions: (**A**) model A; (**B**) model B; and (**C**) model C. (**Left**) side view; (**right**) top view (**top**) and front view (**bottom**) of the model. V represents the fluid’s velocity in μms^−1^.

The final analysis is carried out to study the effects of the trap hole’s position on the cell trapping. Analysis is carried out using a cell trapping model with an *Rh*_Main_/*Rh*_Trap_ ratio of 3.5 and trap hole dimensions of 1, 7, and 2 μm in length, height, and width, respectively. Three different trap hole positions with similar dimensions are analyzed as illustrated in Figure 12, namely models A–C. Cell trapping results demonstrate that all of the models are able to trap cells with an *Rh*_Main_/*Rh*_Trap_ ratio of 3.5 (Figure 8). The streamlines velocity fields produced by the models before cell trapping are focused towards the trap channel. The streamlines show that the fluid stream produced is fully directed toward the trap channel with a similar pattern. The only difference in the streamlines pattern between the models is the position of streams towards the trap hole (Figure 9B,D). The differences could be observed by viewing the streamlines at three different views (Figure 9A). For model A, the streamlines’ focusing could be clearly seen from the top view, where the streamlines’ direction focused on the center of the trap channel (Figure 9A(i)). For models B and C, the streamline focusing could be clearly differentiated by viewing from the front and side (Figure 9C,D(i,ii)), where model B streamlines are focused at the base of the channel while model C are focused at the middle of the trap channel. The findings suggest that a cell trapping model with similar *Rh*_Main_/*Rh*_Trap_ ratio produces similar trapping behavior despite the different trap hole’s positions.

## 3. The Concept of the Model

**Figure 10:**
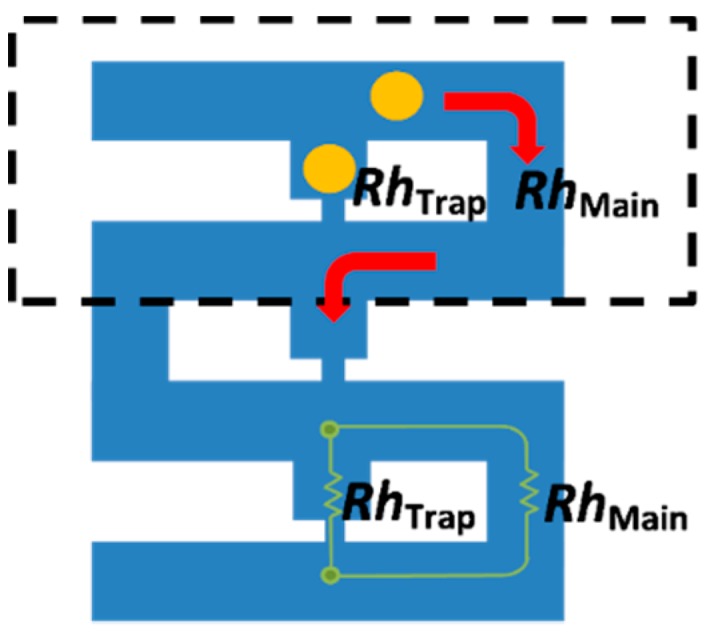
Simple schematic of single-cell trapping channel with the hydrodynamic resistance.

The hydrodynamic trapping concept can be summarized as follows: (a) the trapping channel has a lower *Rh* than the by-passing channel when a trapping site is empty, and will make the particles/cells flow into the trapping stream and directed into the trap; (b) when a bead/cell is trapped, it will act as a plug and will increase the *Rh* along the trap channel drastically; and (c) the main flow will change from the trap channel to the by-pass channel (main channel) and the next particles/cells will be directed to the by-pass stream, passing by the filled trapping site [29]. Figure 10 shows a schematic explanation of the hydrodynamic trapping concept with *Rh*_Trap_ and *Rh*_Main_ representing the flow resistance of trap channel and main channel, respectively. The yellow circle denotes a yeast cell that needs to be trapped.

The Darcy-Weisbach equation is used to determine the pressure drop or pressure difference in a microchannel and solve the continuity and momentum equations for the Hagen-Poiseuille flow problem. From the Hagen–Poiseuille equation, the flow rate (*Q*) can be defined by the following equation:

(1) $$∆P=Q\times Rh=Q\times \left(\frac{C\mu LP^{2}}{A^{3}}\right)$$

where ∆*P* is the pressure drop, *Rh* is the flow resistance of the rectangular channels, *C* is a constant that depends on the aspect ratio (ratio between height and width of the channel), μ is the fluid’s viscosity, and *L*, *P*, and *A* are the length, perimeter, and cross-sectional area of the channel, respectively.

From Equation (1), by approximating that the pressure drop across the trap channel and the main channel are the same (∆*P*_Trap_ = ∆*P*_Main_), the flow rate ratio (*Q*_Trap_/*Q*_main_) or flow resistance ratio (*Rh*_Main_/*Rh*_Trap_) between the trap channel and the main channel can be given as follows [30]:

(2) $$\frac{Q_{\text{Trap}}}{Q_{\text{Main}}}=\frac{Rh_{\text{Main}}}{Rh_{\text{Trap}}}=\left(\frac{C_{\text{Main}}}{C_{\text{Trap}}}\right)\;\left(\frac{L_{\text{Main}}}{L_{\text{Trap}}}\right){\left(\frac{P_{\text{Main}}}{P_{\text{Trap}}}\right)}^{2}{\left(\frac{A_{\text{Trap}}}{A_{\text{Main}}}\right)}^{3}$$

By using a relationship of *A* = *W* × *H* and *P* = *2* (*W* + *H*), where *W* and *H* are the width and height of the channel, respectively, Equation (2) can be defined as:

(3) $$\frac{Q_{\text{Trap}}}{Q_{\text{Main}}}=\frac{Rh_{\text{Main}}}{Rh_{\text{Trap}}}=\left(\frac{C_{\text{Main}}}{C_{\text{Trap}}}\right)\left(\frac{L_{\text{Main}}}{L_{\text{Trap}}}\right){\left(\frac{W_{\text{Main}}+H_{\text{Main}}}{W_{\text{Trap}}+H_{\text{Trap}}}\right)}^{2}{\left(\frac{W_{\text{Trap}}H_{\text{Trap}}}{W_{\text{Main}}H_{\text{Main}}}\right)}^{3}$$

From Equations (2) and (3), it is noted that the flow rates of the trap channel (*Q*_Trap_) and the main channel (*Q*_Main_) are distributed depending on the corresponding *Rh*. For the trap to work, the flow rate along the trap channel must be greater than that of main channel (*Q*_Trap_ > *Q*_Main_). In other words, the flow resistance along the main channel must be greater than that of the trap channel (*Rh*_Main_ > *Rh*_Trap_). Therefore, a single cell can be trapped by manipulating the flow resistance ratio (*Rh*_Main_/*Rh*_Trap_), which is determined by the geometric parameters of the channels.

A single-cell trapping model is developed to produce a finite element single-cell trapping system in which the optimization of a channel’s geometry, dependent on the desired cell size, could be performed. The geometry of the trapping channel is a variable for optimization (see Equation (3)) and subject to the size of cells and the application that will be carried out in the channel after the cells are trapped. An example of a thorough optimization study is presented in this paper using a 5-μm yeast cell model. For other cell sizes, a guideline for designing and optimizing the cell trapping channel is proposed. Firstly the diameter of the viable cells in suspension (floating cells) before cell adhesion occur (for adherent type of cells) should be determined. This is important to determine the range of suitable trap hole sizes. We suggest that the *W*_Hole_ to be less than one third of the cell’s size due to the ability of cells to deform and the flexibility to enter the trap hole instead of being trapped in the trap channel [30]. This could happen, especially to cells that have no cell wall such as human cells. Next, after determining *W*_Hole_, the *H*_Channel_ and *W*_Trap_ have to be optimized. *H*_Channel_ should be bigger than the diameter of the cells to reduce friction between the cell surface and the channel’s wall and to avoid cell squeezing (for applications that do not require cells to be squeezed, e.g., cell culturing, drugs treatment, and cell adhesion study). The optimization of the *L*_Trap_, is dependent on the application of cells after being trapped. Long *L*_Trap_ could cause more than one cell to be trapped if cells in suspension are very near to each other. However, for long-term monitoring of cell behavior for *Tetrahymena thermophila*, a long trap channel is needed to avoid cell from swimming back to the main channel [30]. The trap channel’s geometry size choices are dependent on the application of the trapping platform after the cells/particles are trapped. For example, if adherence cells are used and need to be cultured inside the trapping platform, the *W*_Trap_ should be bigger than the diameter of the cell (viable cells in suspension before adhesion). This is because cells need space for cell adhesion and spreading as the diameter of cells after adhesion will increase depending on culture time. In different applications, individual ciliate protozoan, *Tetrahymena thermophila* [30] need to be trapped and maintained in the trap channel for long-term monitoring of cell behavior. Therefore, no expansion in size is expected after the trapping process and the trap channel’s width does not require space for expansion. In summary, the geometry of channels is a variable (*L*, *H*, and *W*; see Equation (3)) for optimization, subject to the size of cells used and the application that will be carried out after the cells are trapped.

In this single-cell trapping model, cells are introduced into the device through the inlet with an appropriate flow rate and directed to the trap channel by optimizing the channel’s geometry. Trap hole and trap channel geometry are optimized and *L*_Main_ is manipulated to produce an appropriate *Rh* ratio that leads to successful trapping (see Equation (3)). The excess and remaining cells will be directed out through the channel’s outlet by injecting cell’s culture medium. The appropriate channel’s geometry to trap a 5-μm single yeast cell in the specified design is studied. The finite element single-cell trapping model is focusing only on a single trap channel (see dashed box in Figure 1) for geometry optimization due to the complexity and high processing time required for the analysis.

## 4. Simulation Setup

**Figure 11:**
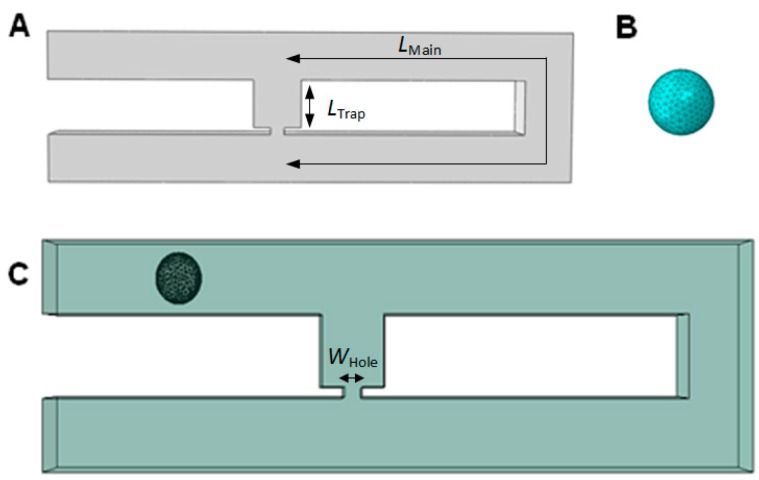
Construction of the finite element model of single-cell trapping system and parts involved: (**A**) Eulerian part (fluid channel’s top view) *L*_Main_ represents the main channel’s length and *L*_Trap_ represents the trap channel’s length; (**B**) 3D deformable part (yeast cell model); (**C**) simulation’s assembly setup (cell is positioned between inlet and trap channel as initial position). *W*_Hole_ represents trap hole’s width.

The analysis is carried out using finite element ABAQUS-FEA™ analysis software, which can perform multiphysics analyses. The single-cell trapping model consists of two different parts, the Eulerian part as the fluid channel and a three-dimensional (3D) deformable part as the sphere-shaped elastic yeast cell model (Figure 11A,B). The fluid consists of two microchannels, the main channel (loop channel) and a trap channel with a rectangular trap hole placed in the center, at the edge of the trap channel. The microchannel is modeled as 3D Eulerian explicit EC3DR and an eight-node linear Eulerian brick element part assigned with water properties (density, equation of state, and viscosity). A sphere-shaped yeast cell (5 μm in diameter) is modeled as an elastic 3D standard solid deformable C3D8R and an eight-node linear brick 3D part with the yeast properties (Young’s modulus, Poisson’s ratio, and density) obtained from literature [31,32,33,34,35,36,37,38].

Figure 11C shows the assembly setup with a yeast cell positioned in the main channel, near the channel’s inlet (left). The parts are assembled to develop the finite element model for the proposed system (Figure 11C). The initial position of the cell is fixed (same distance between cell and trap channel) for all models. Interaction between cell and water is set as general contact with rough tangential behavior and the interaction between cell surface and channel’s wall is set as frictionless. The fluid channel and cell are meshed using hexahedron mesh types. Total mesh elements for the cell trapping model ranged from 10,627 to 22,485 elements. No-inflow and non-reflecting outflow Eulerian boundary conditions are applied to the channel’s wall. A constant inflow velocity of 0.5 μms^−1^ is applied to the inlet and atmosphere pressure is applied to the outlet of the channel.

**Figure 12:**
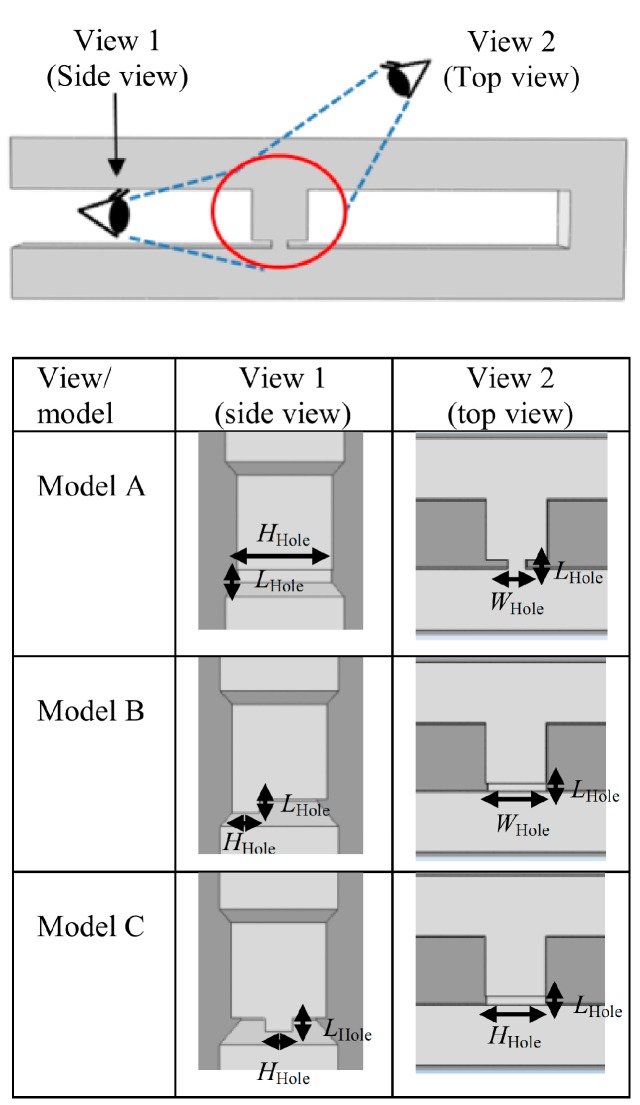
The views for three cell trapping models with the same trap hole size (2, 7 and 7 μm for *W*_Hole_, *L*_Hole_, and *H*_Hole_, respectively) at different positions.

The simulation analysis could be divided into four parts: the verification of the hydrodynamic trapping concept, the effects of *Rh*_Main_/*Rh*_Trap_ ratio in cell trapping, the optimization of the trap channel’s length, and the effects of the trap hole’s position. For the verification of the hydrodynamic trapping concept, a model with a trap hole’s width of 2.0 μm is used for the analysis. To study the effects of *Rh*_Main_/*Rh*_Trap_ ratio in cell trapping, various *L*_Main_ ranging from 46 to 268 μm (Figure 11A) and *W*_Hole_ ranging from 1.0 to 3.5 μm (Figure 2C) with fixed *L*_Hole_ of 1 μm are applied to obtain the appropriate *Rh*_Main_/*Rh*_Trap_ ratio for cell trapping. The height of main channel, trap channel, and trap hole are uniform (*H*_Channel_) and were tested in the range of 6–9 μm and set to be 7 μm throughout the analysis. For trap channel length (*L*_Trap_) optimization, various trap channel lengths from 3 to 9 μm and a fixed trap channel width of 7 μm are used, with three yeast cells in the analysis. Lastly, to study the effects of trap hole’s position, three different positions for similar trap hole’s dimensions are studied to observe the ability of the model for cell trapping. Figure 12 shows the different views for the three different positions, represented by models A, B, and C.

## 5. Conclusions

This study presents the finite element model of single-cell trapping inside microfluidic channel. This single-cell trapping system is constructed using Abaqus-FEA™ software. A guideline to design and optimize single-cell trapping model is proposed and the example of a thorough optimization analysis is carried out using a yeast cell model. The results show that the finite element model is able to trap a single cell inside the fluidic environment. The fluid velocity profile and streamline plots of successful and unsuccessful single yeast cell trapping are presented according to the hydrodynamic concept. This cell trapping model is able to isolate an individual yeast cell inside a fluidic environment, thus providing a platform for further single-cell mechanical or biological study. Single-cell manipulation such as chemical and biophysical treatments and also mechanical characterization could be performed inside the microfluidic channel using this system. The single-cell trapping model can be a significant important guideline in designing a new chip for biomedical applications.

## References

[1] Johann R.M. (2006). Cell Trapping in Microfluidic Chips. Anal. Bioanal. Chem..

[2] Lee G.-H., Kim S.-H., Kang A., Takayama S., Lee S.-H., Park J.Y. (2015). Deformable L-shaped microwell array for trapping pairs of heterogeneous cells. J. Micromech. Microeng..

[3] Sun T., Kovac J., Voldman J. (2014). Image-based single-cell sorting via dual-photopolymerized microwell arrays. Anal. Chem..

[4] Rettig J.R., Folch A. (2005). Large-scale single-cell trapping and imaging using microwell arrays. Anal. Chem..

[5] Tang J., Peng R., Ding J. (2010). The Regulation of Stem cell differentiation by cell-cell contact on micropatterned material surfaces. Biomaterials.

[6] Doh J., Kim M., Krummel M.F. (2010). Cell-laden microwells for the study of multicellularity in lymphocyte fate decisions. Biomaterials.

[7] Chen N.-C., Chen C.-H., Chen M.-K., Jang L.-S., Wang M.-H. (2014). Single-cell trapping and impedance measurement utilizing dielectrophoresis in a parallel-plate microfluidic device. Sens. Actuators B Chem..

[8] Sen M., Ino K., Ramon-Azcon J., Shiku H., Matsue T. (2013). Cell pairing using a dielectrophoresis-based device with interdigitated array electrodes. Lab Chip.

[9] Voldman J., Gray M.L., Toner M., Schmidt M.A. (2002). A Microfabrication-based dynamic array cytometer. Anal. Chem..

[10] Thomas R.S., Morgan H., Green N.G. (2009). Negative DEP traps for single cell immobilisation. Lab Chip.

[11] Gray D.S., Tan J.L., Voldman J., Chen C.S. (2004). Dielectrophoretic registration of living cells to a microelectrode array. Biosens. Bioelectron..

[12] Chen Y.-C., Allen S.G., Ingram P.N., Buckanovich R., Merajver S.D., Yoon E. (2015). Single-cell migration chip for chemotaxis-based microfluidic selection of heterogeneous cell populations. Sci. Rep..

[13] Jin D., Deng B., Li J.X., Cai W., Tu L., Chen J., Wu Q., Wang W.H. (2015). A Microfluidic device enabling high-efficiency single cell trapping. Biomicrofluidics.

[14] Benavente-Babace A., Gallego-Pérez D., Hansford D.J., Arana S., Pérez-Lorenzo E., Mujika M. (2014). Single-cell trapping and selective treatment via co-flow within a microfluidic platform. Biosens. Bioelectron..

[15] Kim J., Erath J., Rodriguez A., Yang C. (2014). A High-efficiency microfluidic device for size-selective trapping and sorting. Lab Chip.

[16] Lee P.J., Hung P.J., Shaw R., Jan L., Lee L.P. (2005). Microfluidic application-specific integrated device for monitoring direct cell-cell communication via gap junctions between individual cell pairs. Appl. Phys. Lett..

[17] Frimat J.-P., Becker M., Chiang Y.-Y., Marggraf U., Janasek D., Hengstler J.G., Franzke J., West J. (2011). A microfluidic array with cellular valving for single cell co-culture. Lab Chip.

[18] Kim H., Lee S., Kim J. (2012). Hydrodynamic trap-and-release of single particles using dual-function elastomeric valves: design, fabrication, and characterization. Microfluid. Nanofluid..

[19] Arakawa T., Noguchi M., Sumitomo K., Yamaguchi Y., Shoji S. (2011). High-throughput single-cell manipulation system for a large number of target cells. Biomicrofluidics.

[20] Kobel S., Valero A., Latt J., Renaud P., Lutolf M. (2010). Optimization of microfluidic single cell trapping for long-term on-chip culture. Lab Chip.

[21] Hong S., Pan Q., Lee L.P. (2012). Single-cell level co-culture platform for intercellular communication. Integr. Biol..

[22] Shi W., Qin J., Ye N., Lin B. (2008). Droplet-based microfluidic system for individual caenorhabditis elegans assay. Lab Chip.

[23] Di Carlo D., Aghdam N., Lee L.P. (2006). Single-cell enzyme concentrations, kinetics, and inhibition analysis using high-density hydrodynamic cell isolation arrays. Anal. Chem..

[24] Skelley A.M., Kirak O., Suh H., Jaenisch R., Voldman J. (2009). Microfluidic control of cell pairing and fusion. Nat. Methods.

[25] Di Carlo D., Wu L.Y., Lee L.P. (2006). Dynamic single cell culture array. Lab Chip.

[26] Tan W.-H., Takeuchi S. (2007). A Trap-and-release Integrated microfluidic system for dynamic microarray applications. Proc. Natl. Acad. Sci. USA.

[27] Chung K., Rivet C.A., Kemp M.L., Lu H., States U. (2011). Imaging single-cell signaling dynamics with a deterministic high-density single-cell trap array. Anal. Chem..

[28] Khalili A.A., Basri M.A.M., Ahmad M.R. (2014). Simulation of single cell trapping via hydrodynamic manipulation. J. Teknol..

[29] Teshima T., Ishihara H., Iwai K., Adachi A., Takeuchi S. (2010). A dynamic microarray device for paired bead-based analysis. Lab Chip.

[30] Kumano I., Hosoda K., Suzuki H., Hirata K., Yomo T. (2012). Hydrodynamic trapping of tetrahymena thermophila for the long-term monitoring of cell behaviors. Lab Chip.

[31] Gervais T., El-Ali J., Günther A., Jensen K.F. (2006). Flow-induced deformation of shallow microfluidic channels. Lab Chip.

[32] Bryan A.K., Goranov A., Amon A., Manalis S.R. (2010). Measurement of mass, density, and volume during the cell cycle of yeast. Proc. Natl. Acad. Sci. USA.

[33] Ahmad M.R., Nakajima M., Kojima S., Homma M., Fukuda T. (2008). The Effects of cell sizes, environmental conditions, and growth phases on the strength of individual W303 yeast cells inside ESEM. IEEE Trans. Nanobiosci..

[34] Smith A.E., Zhang Z., Thomas C.R., Moxham K.E., Middelberg A.P. (2000). The Mechanical properties of saccharomyces cerevisiae. Proc. Natl. Acad. Sci. USA.

[35] Stenson J.D., Thomas C.R., Hartley P. (2009). Modelling the mechanical properties of yeast cells. Chem. Eng. Sci..

[36] Stenson J.D., Hartley P., Wang C., Thomas C.R. (2011). Determining the mechanical properties of yeast cell walls. Biotechnol. Prog..

[37] Burg T.P., Godin M., Knudsen S.M., Shen W., Carlson G., Foster J.S., Babcock K., Manalis S.R. (2007). Weighing of Biomolecules, Single Cells and Single Nanoparticles in Fluid. Nature.

[38] Lee J., Chunara R., Shen W., Payer K., Babcock K., Burg T.P., Manalis S.R. (2011). Suspended microchannel resonators with piezoresistive sensors. Lab Chip.

